# Supplementary material for: Actin cytoskeleton deregulation confers midostaurin resistance in FLT3-mutant acute myeloid leukemia
Source: Commun Biol. 2021 Jun 25;4:799. doi: 10.1038/s42003-021-02215-w (PMC8233337; doi:10.1038/s42003-021-02215-w)
Supplement: Supplementary file 2 — Supplementary Information [file 42003_2021_2215_MOESM2_ESM.pdf]

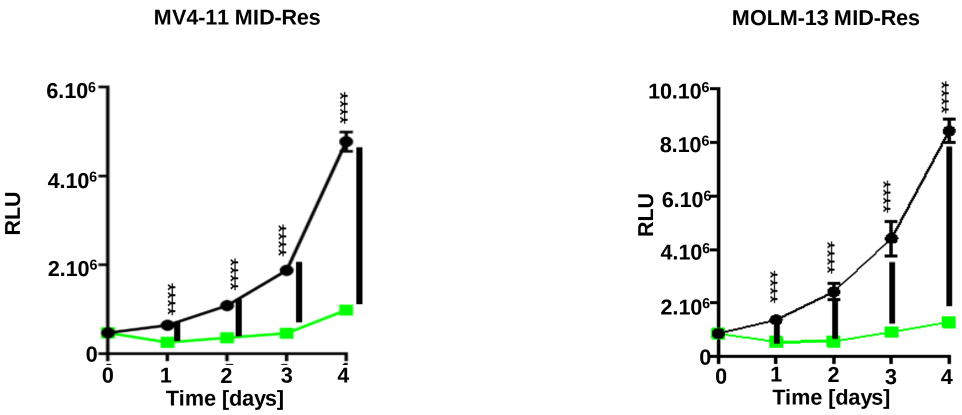

Fig. S1. FLT3 knock down reduces the cell growth of MV4-11 and MOLM-13 MID-Res cells. Proliferation analysis in FLT3 KD MV4-11/MOLM-13 MID-Res (green) compared to scramble MID-Res cells (black) during 4 days. Data are shown as means ± SDs (error bars) from three independent experiments.

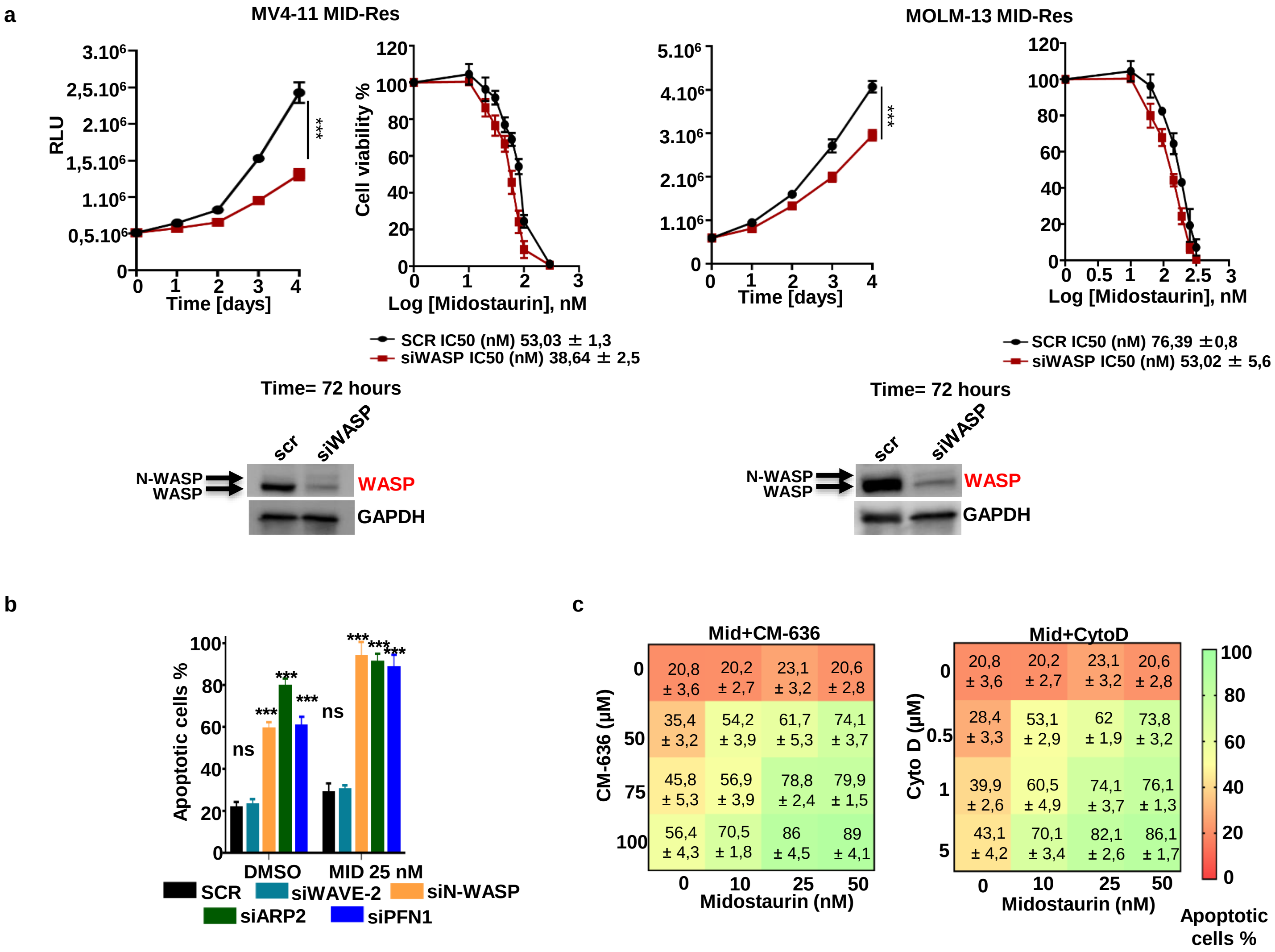

Fig. S2. FLT3 dependent RAC1 activation promotes dysregulation of actin polymerization regulators in MOLM-13 Midostaurin resistance cells. A) Proliferation, Mid IC50 and WASP protein expression analysis in MV4-11/MOLM-13 MID-Res WASP KD compared to scramble MID-Res. B) Apoptosis analysis in MV4-11 MID-Res N-WASP KD, WAVE2 KD, ARP2/3 complex and PFN1 KD compared to scramble MID-Res cells -/+ 25 nM Mid during 24h C) Apoptosis analysis in MV4-11 MID-Res treated with Mid, CM-636 and Cytochlasin D alone/combination during 48 hours. D) Cell viability and Mid IC50 analysis in CFL1 KD MV4-11 parental cells compared to scramble MV4-11 treated with different Mid doses. CFL1 protein expression analysis in CFL1 KD MV4-11 parental cells compared to scramble MV4-11. Data are shown as means ± SDs (error bars) from three independent experiments.

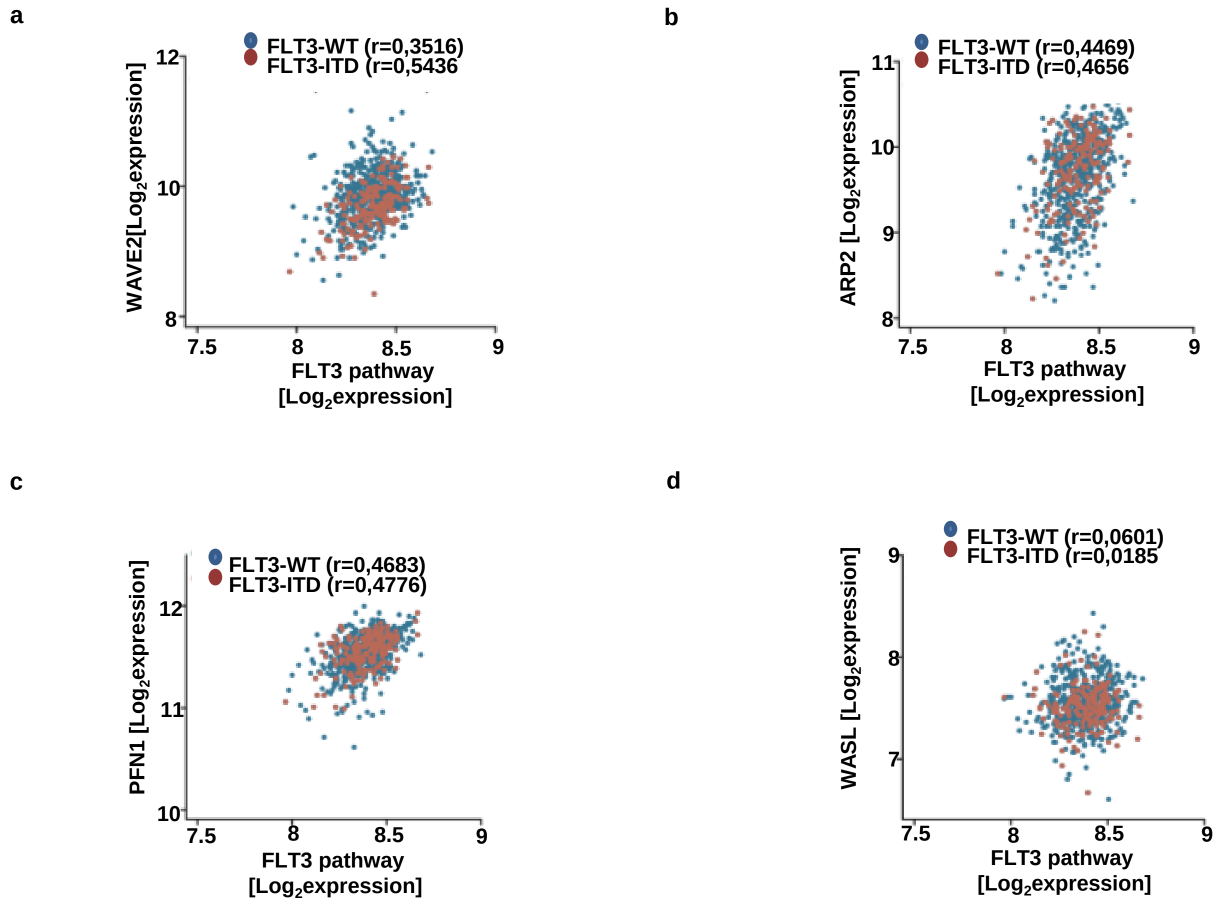

Fig. S3. Correlation analysis between actin polymerization inducers and FLT3 signaling. A) Correlation analysis between WAVE2 and FLT3 pathway genes expression in 639 AML patients. B) Correlation analysis between ARP2 and FLT3 pathway genes expression in 639 AML patients. C) Correlation analysis between PFN1 and FLT3 pathway genes expression in 639 AML patients. D) Correlation analysis between N-WASP and FLT3 dependent pathway genes expression in 639 AML patients.

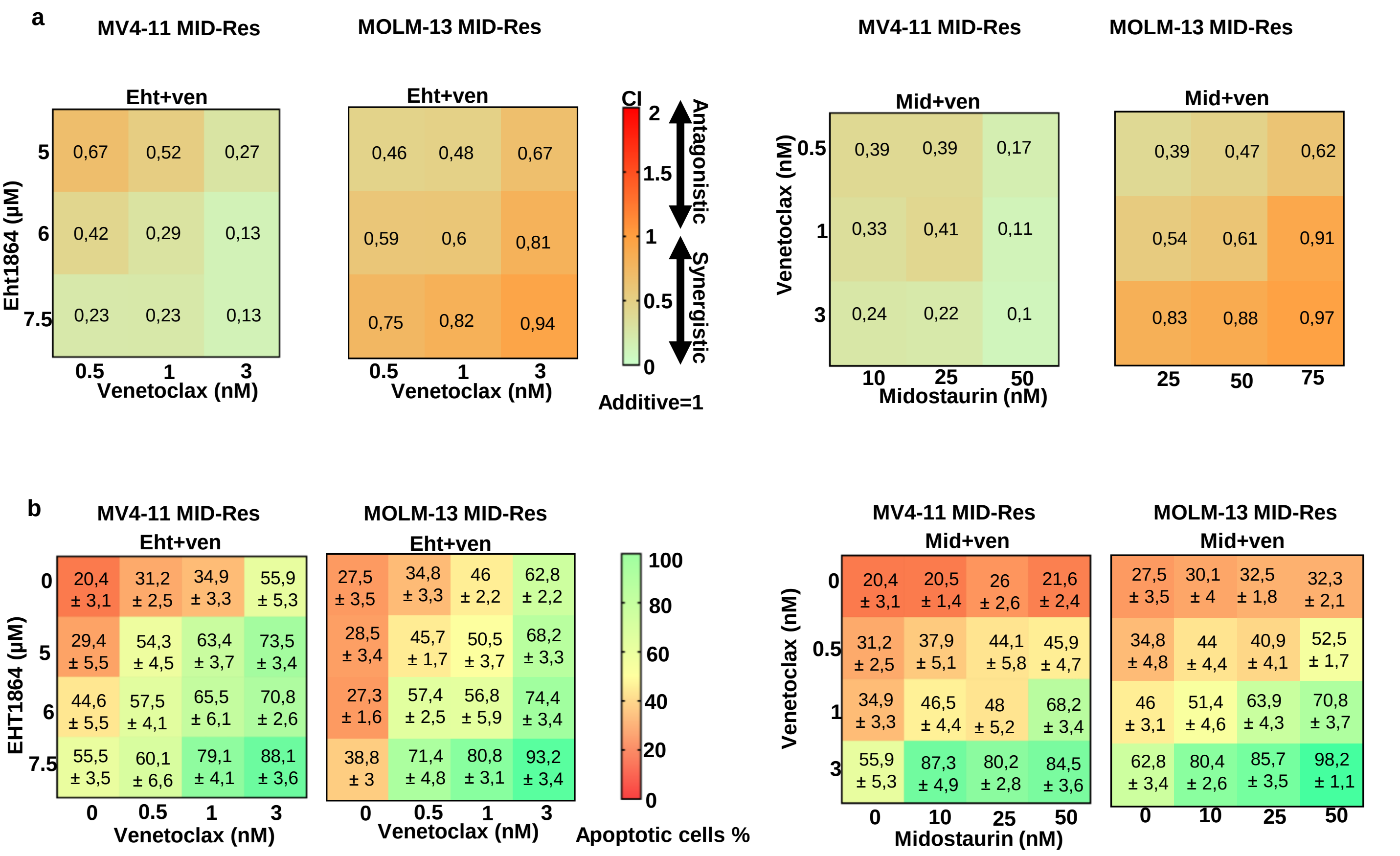

Fig. S4. The cell viability and MCL1/BCL-2 protein expression analysis in MOLM-13 Midostaurin resistant cells treated with the combinations between Eht1864 + Venetolax and Midostaurin + Venetoclax. A) Synergy analysis in MV4-11/MOLM-13 MID-Res treated with Mid, Ve and Eht alone/combination during 48 hours. C) Apoptosis analysis in MOLM-13 and MV4-11 MID-Res treated with Mid, Eht and Ve alone/combination during 48 hours.

Unprocessed Western blots

Figure 1B

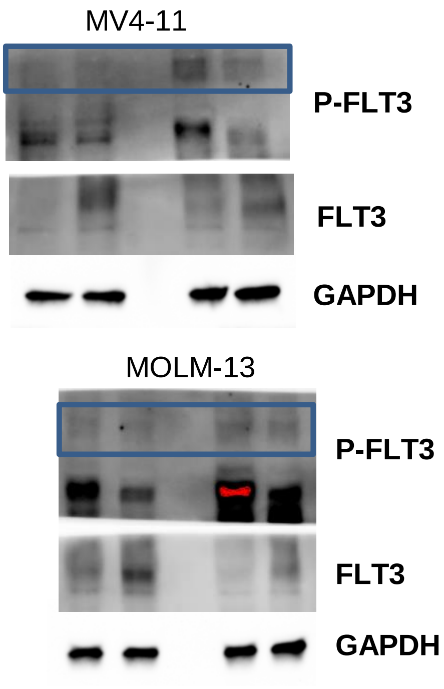

Figure 1F

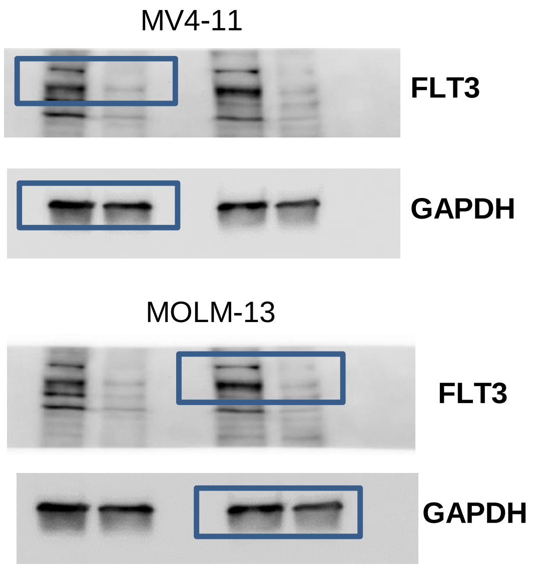

Figure 2C

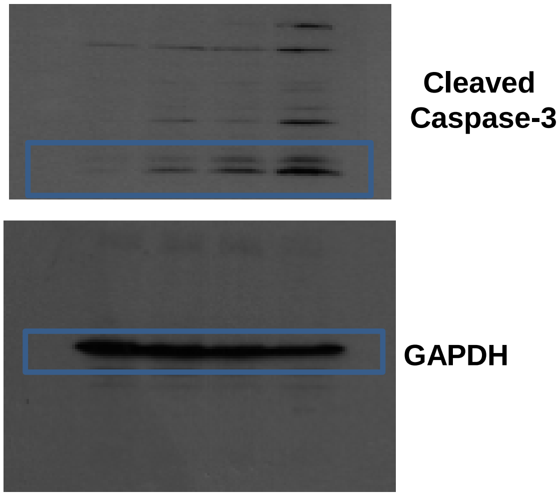

Fig. S5. Unprocessed Western blots .

Unprocessed Western blots

Figure 3 A (MID-sens/MID-Res)

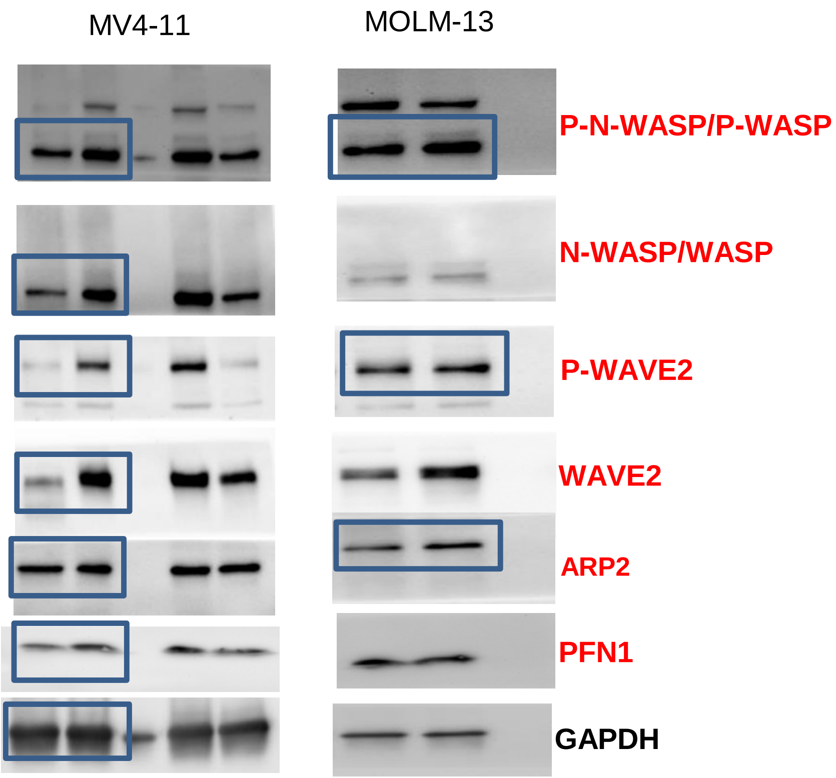

Figure 3 A (-/+ MID)

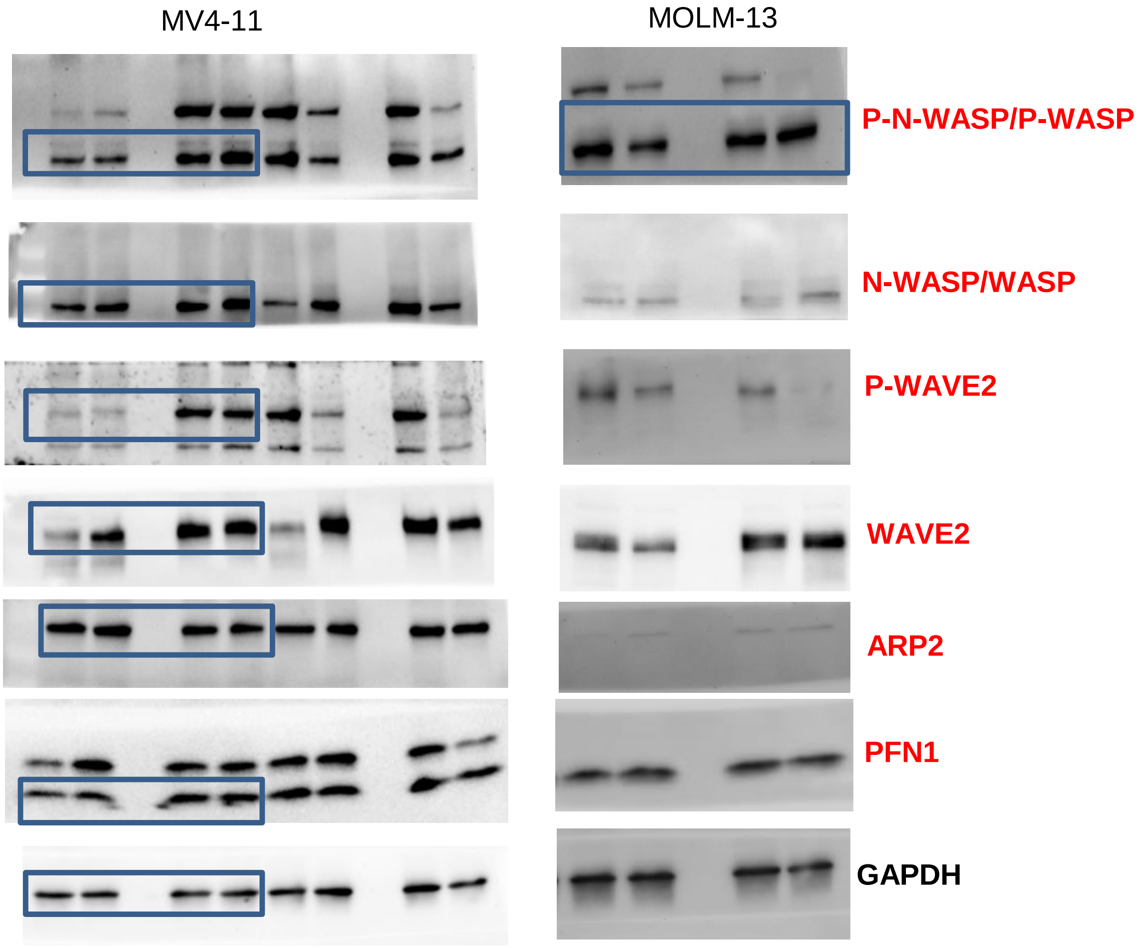

Fig. S6. Unprocessed Western blots

Unprocessed Western blots

Figure 3 B

MV4-11 (FLT3 KD vs scr)

MOLM-13 (FLT3 KD vs scr)

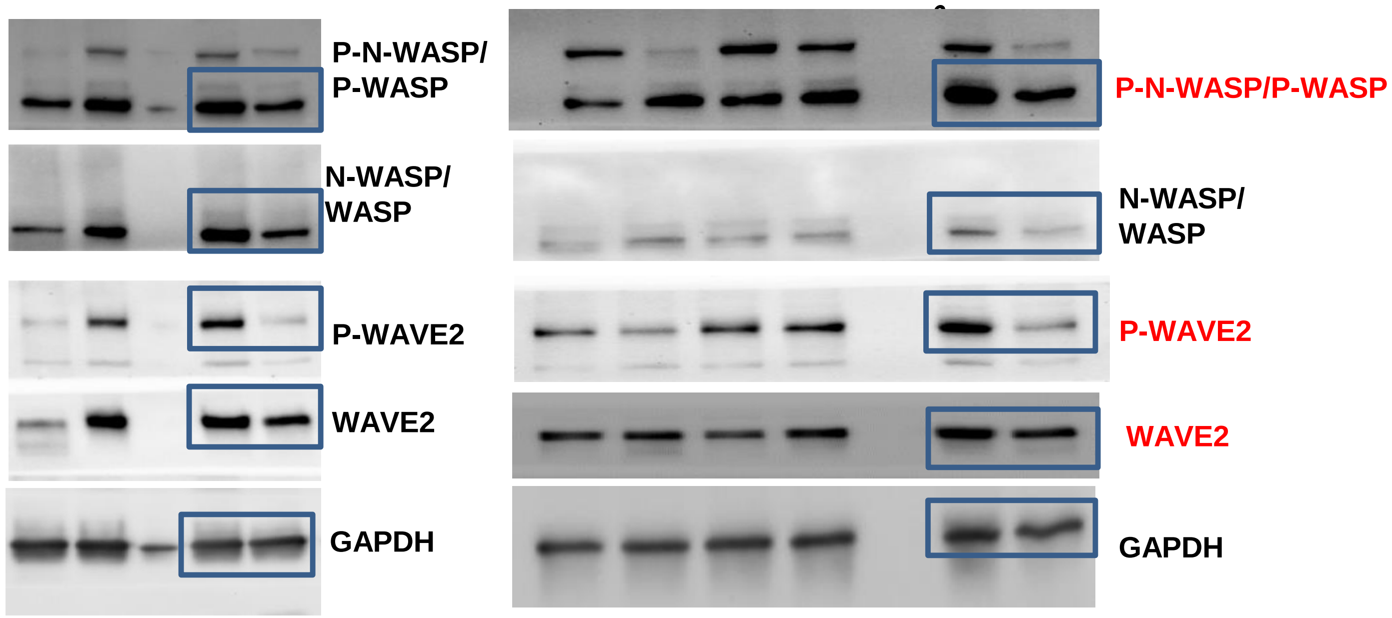

Figure 3 B

(EHT1864+Midostaurin combination)

MV4-11

MOLM-13

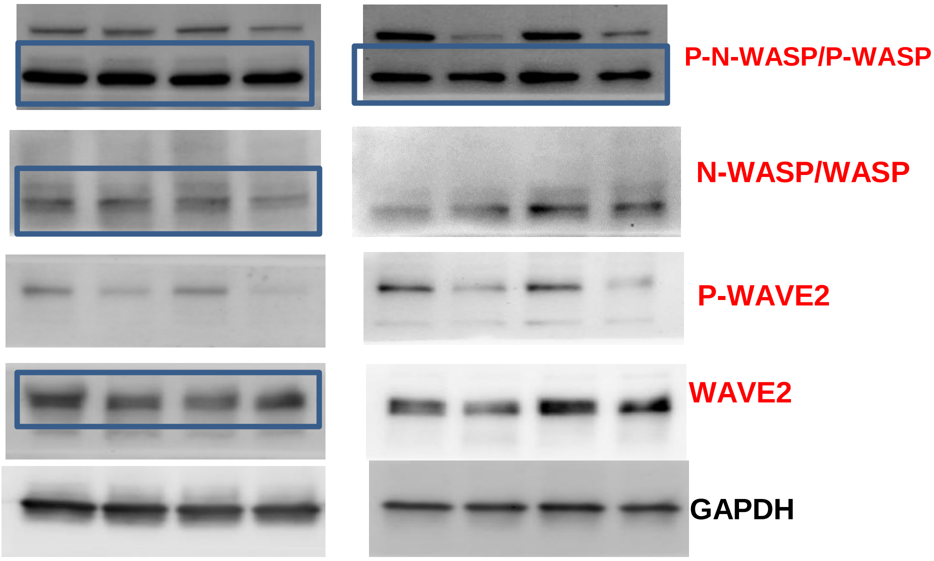

Figure 3 D

MV4-11

MOLM-13

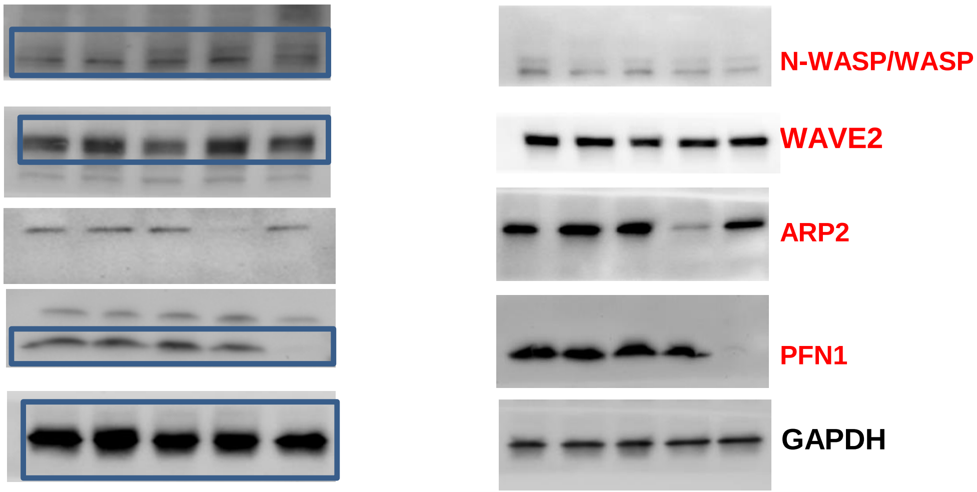

Fig. S7. Unprocessed Western blots.

Unprocessed Western blots

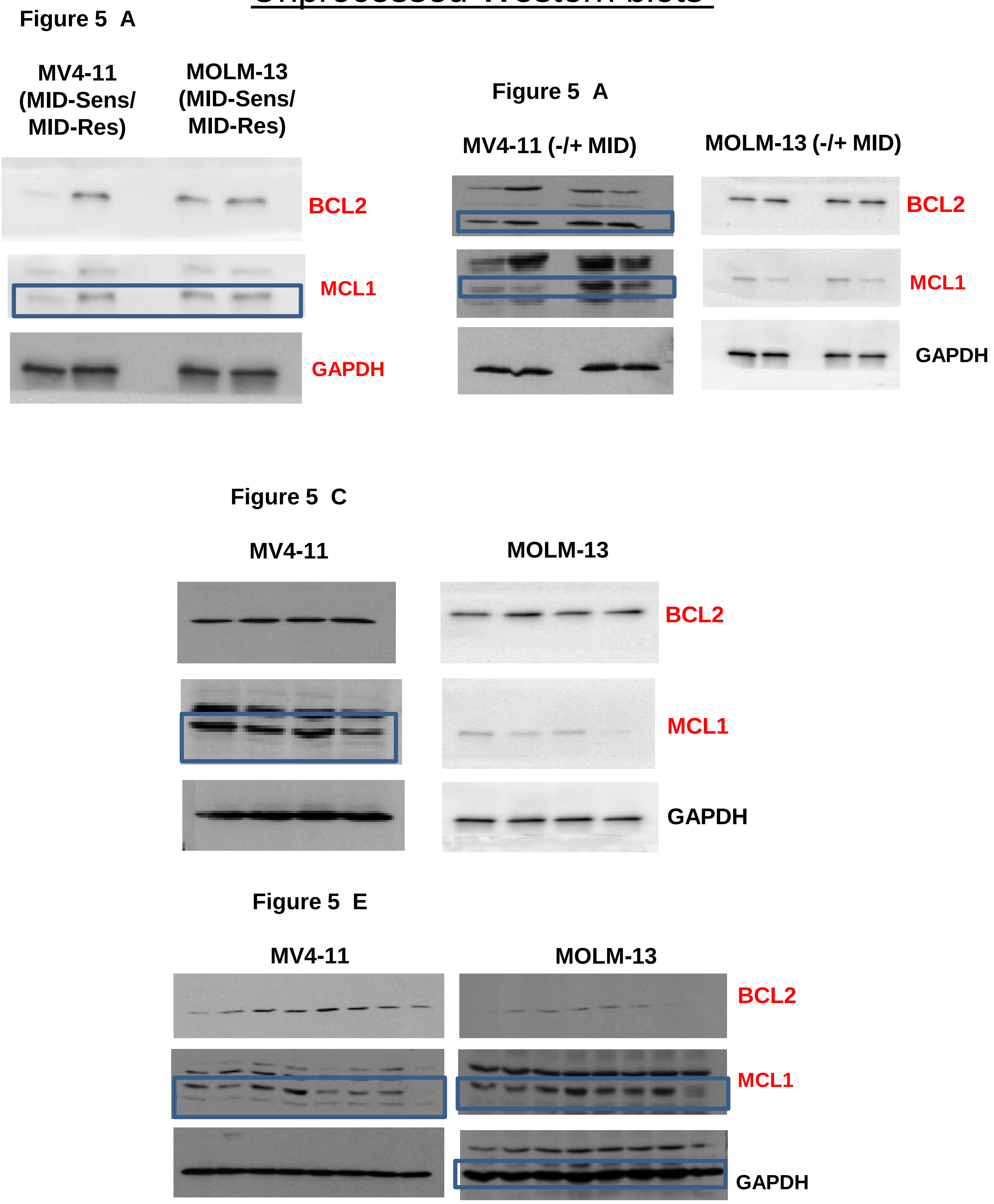

Fig. S8. Unprocessed Western blots.

Patient	Date of birth	Molecular genetic	Initial therapy	Therapy response	The blast % after treatment (Bone Marrow)
REF001	16.09.1947	FLT3-TKD D835, NPM1	Cytarabine +Daunorubicin+Midostaurin	Non-response	50%
REF002	18.06.1948	FLT3-ITD	Cytarabine +Daunorubicin+Midostaurin	Non-response	60%

Table S1. AML primary samples information.
