# Supplementary material for: Actin cytoskeleton deregulation confers midostaurin resistance in FLT3-mutant acute myeloid leukemia
Source: Commun Biol. 2021 Jun 25;4:799. doi: 10.1038/s42003-021-02215-w (PMC8233337; doi:10.1038/s42003-021-02215-w)
Supplement: Supplementary file 3 — Description of Additional Supplementary Files [file 42003_2021_2215_MOESM3_ESM.pdf]

Description of Additional Supplementary Files

File Name: Supplementary Movie 1

Description: Adhesion forces measurement between MV4-11 MID-Sens and MSCs HS-5. MV4-11 MID-Sens are approached with the cantilever until it pressed the surface of a cell. The cell is immobilized at the cantilever aperture by applying an under pressure until complete detachment from the stromal layer.

File Name: Supplementary Movie 2

Description: Adhesion forces measurement between MV4-11 MID-Res and HS-5. MV4-11 MID-Res are approached with the cantilever until it pressed the surface of a cell. The cell was immobilized at the cantilever aperture by applying an under pressure until complete detachment from the stromal layer.

File Name: Supplementary Movie 3

Description: Co-culture of MV4-11 MID-Sens and HS-5 cells shaking the plate. The video shows the weak adhesion forces between MID-Sens and HS-5 cells.

File Name: Supplementary Movie 4

Description: Co-culture of MV4-11 MID-Res and HS-5 cells shaking the plate. The video shows the strong adhesion forces between MID-Sens and HS-5 cells.

File Name: Supplementary Movie 5

Description: Co-culture of MV4-11 MID-Res treated during 12 hours with Eht1864 and HS-5 cells shaking the plate. The video shows that MID-Res cells under Rac1 inhibitor treatment are start detaching from HS-5 cells.

File name: Supplementary Data 1

Description: The source data behind the graphs in the paper.
