# Supplementary material for: Actin cytoskeleton deregulation confers midostaurin resistance in FLT3-mutant acute myeloid leukemia
Source: Commun Biol. 2021 Jun 25;4:799. doi: 10.1038/s42003-021-02215-w (PMC8233337; doi:10.1038/s42003-021-02215-w)
Supplement: Supplementary file 10 — Reporting Summary [file 42003_2021_2215_MOESM10_ESM.pdf]

nature research

Corresponding author(s): Martin Kortuem
Last updated by author(s): 2021.02.09

Reporting Summary

Nature Research wishes to improve the reproducibility of the work that we publish. This form provides structure for consistency and transparency in reporting. For further information on Nature Research policies, see our Editorial Policies and the Editorial Policy Checklist.

Statistics

For all statistical analyses, confirm that the following items are present in the figure legend, table legend, main text, or Methods section.

n/a	Confirmed	
☐	☒	The exact sample size (*n*) for each experimental group/condition, given as a discrete number and unit of measurement
☐	☒	A statement on whether measurements were taken from distinct samples or whether the same sample was measured repeatedly
☐	☒	The statistical test(s) used AND whether they are one- or two-sided<br>*Only common tests should be described solely by name; describe more complex techniques in the Methods section.*
☒	☐	A description of all covariates tested
☐	☒	A description of any assumptions or corrections, such as tests of normality and adjustment for multiple comparisons
☐	☒	A full description of the statistical parameters including central tendency (e.g. means) or other basic estimates (e.g. regression coefficient) AND variation (e.g. standard deviation) or associated estimates of uncertainty (e.g. confidence intervals)
☐	☒	For null hypothesis testing, the test statistic (e.g. *F*, *t*, *r*) with confidence intervals, effect sizes, degrees of freedom and *P* value noted<br>*Give P values as exact values whenever suitable.*
☒	☐	For Bayesian analysis, information on the choice of priors and Markov chain Monte Carlo settings
☒	☐	For hierarchical and complex designs, identification of the appropriate level for tests and full reporting of outcomes
☒	☐	Estimates of effect sizes (e.g. Cohen's *d*, Pearson's *r*), indicating how they were calculated

Our web collection on statistics for biologists contains articles on many of the points above.

Software and code

Policy information about availability of computer code

Data collection	Correlation analysis between the expression of actin polymerization inducers and FLT3, signaling in AML patients, Meta information and gene expression data of 639 AML patients were obtained from ArrayExpress (E-MTAB3444).
Data analysis	Raw data were RMA normalized using the {affy} package and multiple probes per gene were aggregated by mean. Flow cytometry data were analyzed by FlowJo V.10.6. Western blots were quantified by Image J software. GraphPad Prism 7.0.

For manuscripts utilizing custom algorithms or software that are central to the research but not yet described in published literature, software must be made available to editors and reviewers. We strongly encourage code deposition in a community repository (e.g. GitHub). See the Nature Research guidelines for submitting code & software for further information.

Data

Policy information about availability of data

All manuscripts must include a data availability statement. This statement should provide the following information, where applicable:

- Accession codes, unique identifiers, or web links for publicly available datasets
- A list of figures that have associated raw data
- A description of any restrictions on data availability

Meta information and gene expression data of 639 AML patients were obtained from ArrayExpress (E-MTAB3444). FLT3 target genes (n=61) were extracted from PathCards (https://pathcards.genecards.org/; using the “FLT3 signaling” SuperPath.

Field-specific reporting

Please select the one below that is the best fit for your research. If you are not sure, read the appropriate sections before making your selection.

☒ Life sciences ☐ Behavioural & social sciences ☐ Ecological, evolutionary & environmental sciences

For a reference copy of the document with all sections, see nature.com/documents/nr-reporting-summary-flat.pdf

Life sciences study design

All studies must disclose on these points even when the disclosure is negative.

Sample size	PBMCs from healthy donors n=6.<br>AML Blast cells from AML patients bone marrow n=2<br>Correlation analysis between the expression of actin polymerization inducers and FLT3, signaling in AML patients n=639.
Data exclusions	No data exclusions.
Replication	Data are shown as means ± SDs (error bars) from three independent experiments.
Randomization	Samples allocation was random in the experimental groups.
Blinding	Blinding was not possible, we had to find out the minimal dose of triple drug combination that induced cell death in AML primary samples/cell lines and then test its effect in PBMCs obtained from healthy donors.

Reporting for specific materials, systems and methods

We require information from authors about some types of materials, experimental systems and methods used in many studies. Here, indicate whether each material, system or method listed is relevant to your study. If you are not sure if a list item applies to your research, read the appropriate section before selecting a response.

Materials & experimental systems

n/a	Involved in the study	
☐	☒	Antibodies
☐	☒	Eukaryotic cell lines
☒	☐	Palaeontology and archaeology
☒	☐	Animals and other organisms
☒	☐	Human research participants
☒	☐	Clinical data
☒	☐	Dual use research of concern

Methods

n/a	Involved in the study	
☒	☐	ChIP-seq
☐	☒	Flow cytometry
☒	☐	MRI-based neuroimaging

Antibodies

Antibodies used	Anti-Phospho Tyrosin 969 FLT3, Anti-ARP2, anti-N-WASP, anti-WAVE2, anti-PFN1, anti–MCL1, anti–BCL-2, and anti-GAPDH antibodies were purchased from Cell Signaling Technology; The HRP-conjugated secondary antibody were from Cell Signaling Technology. Moreover, it was used anti-P-N-WASP [Ser484/Ser485] and anti-P-WAVE2 [Ser343] from Merck.
Validation	All the antibodies were validated by the company and our siRNAs experiments confirmed their specificity. Regarding N-WASP antibody, the company states that just detects N-WASP. But we have shown by western blot and siRNAs experiments that this antibody detects N-WASP and WASP proteins. It has been already reported to Cell Signaling company.

Eukaryotic cell lines

Policy information about cell lines

Cell line source(s)	ATCC and DSMZ
Authentication	It was carried out cell lines Authentication.
Mycoplasma contamination	The cell lines were tested for mycoplasma contamination monthly
Commonly misidentified lines (See ICLAC register)	*Name any commonly misidentified cell lines used in the study and provide a rationale for their use.*

Flow Cytometry

Plots

Confirm that:

- [x] The axis labels state the marker and fluorochrome used (e.g. CD4-FITC).
- [x] The axis scales are clearly visible. Include numbers along axes only for bottom left plot of group (a 'group' is an analysis of identical markers).
- [x] All plots are contour plots with outliers or pseudocolor plots.
- [x] A numerical value for number of cells or percentage (with statistics) is provided.

Methodology

Sample preparation	1x106 cells in 100µl FACS Buffer (1X PBS + 1% FCS + 5mM HEPES) were stained with 20µl of PE-FLT3 (the optimum concentration was determined after titrating the antibody) for 30 minutes at 4°C. After washing the cells, a viability dye (1.1000) was used (Cat. No. L34960, ThermoFisher Scientific, Erlangen, Germany) for 30 minutes at room temperature. Cells were washed and resuspended in 300µl FACS Buffer
Instrument	the CytoFLEX flow cytometer (Beckman Coulter).
Software	FlowJo V.10.6
Cell population abundance	n/a.
Gating strategy	The gating strategy consisted of debris removal (FSC/SSC), double discrimination (FSC-A/H), 24 dead cell exclusion (APC-) and FLT3+.

- [ ] Tick this box to confirm that a figure exemplifying the gating strategy is provided in the Supplementary Information.
